# Emerging role of exosome-derived long non-coding RNAs in tumor microenvironment

**DOI:** 10.1186/s12943-018-0831-z

**Published:** 2018-04-20

**Authors:** Zhenqiang Sun, Shuaixi Yang, Quanbo Zhou, Guixian Wang, Junmin Song, Zhen Li, Zhiyong Zhang, Jizhong Xu, Kunkun Xia, Yuan Chang, Jinbo Liu, Weitang Yuan

**Affiliations:** Department of Anorectal Surgery, The First Affiliated Hospital of Zhengzhou University, Zhengzhou, 450052 Henan China

**Keywords:** Exosomes, Long non-coding RNA, Tumor microenvironment, Cancer, Biomarker

## Abstract

Exosomes are extracellular vesicles released by many cell types and have been attributed for their roles in many diseases including cancer. Exosomes secreted by tumor cells and stromal cells are critical mediators of intercellular communication in tumor microenvironments. Long noncoding RNAs (lncRNAs) are selectively sorted into exosomes and can regulate cancer onset and progression in a variety of ways. In this review, we summarize the characteristics of exosomal lncRNAs and their dysregulation in multiple types of cancer. We provide an overview of current research on exosomal lncRNAs in tumor microenvironments, especially the functions of exosomal lncRNAs in regulating tumor biology. A deeper understanding of the role of exosomal lncRNAs in the tumor microenvironment may help provide new diagnostic and prognostic markers for cancer.

## Background

Extracellular vesicles (EVs) are small membranous vesicles that are secreted from numerous cell types, including apoptotic bodies, microvesicles, and exosomes [1]. Exosomes are generated inside multivesicular endosomes or multivesicular bodies (MVBs) and are secreted when these compartments fuse with the plasma membrane [2]. Exosomes facilitate intercellular communication by transporting intracellular components such as protein, RNA and DNA [3]. These components of exosomes are functional in the recipient cells and highly variable depending on the origin cells, and cells can produce different exosomes under different physiological and pathological conditions [4]. In 1981, it was reported that exosomes were 40–150 nm in diameter and could be isolated from various normal and tumor cells [5]. Numerous studies have shown that exosomes are critical mediators of intercellular communication between tumor cells and stromal cells in local and distant microenvironments [6]. The surface molecules of exosomes allow them to target recipient cells [7, 8]. Once attached to a target cell, exosomes induce signaling via receptor-ligand interactions or can be internalized by endocytosis and/or phagocytosis [9]. On the other hand, exosomes even fuse with the membrane of the target cell to deliver their contents into its cytosol. Exosomes can be isolated from diverse biofluids, such as blood [10], urine [11] and saliva [12]. Different techniques have been used to isolate exosomes, such as ultracentrifugation, density gradients, immunoaffinity, and commercial kits [13]. Furthermore, several methods have been applied to identify exosomes, such as electron microscopy, flow cytometry and western blot analysis [14]. Finally, high-throughput sequencing of clinical samples has been performed to analyze the differential expression of exosomal lncRNAs, which may be potential markers for cancer diagnosis.

According to the origin of the exosomes in the tumor microenvironment, exosomes can be divided into tumor cell secretions and stromal cell secretions. Cancer cells aberrantly secrete large amounts of exosomes to reflect the phenotypic state of stromal cells [15]. As tumors progress, the cargo released in exosomes also dynamically changes, thereby promoting cancer progression. Once taken in by recipient cells, tumor-derived exosomes (TEX) contribute to the crosstalk between cancer cells and stromal cells in the tumor microenvironment, such as fibroblasts [16], endothelial cells [17], and immune cells [18]. For instance, cancer cells can inhibit effector functions and induce apoptosis of various immune cells by releasing components of the TEX cargo, such as TGF-β [19], NKG2D [20], miR-203 [21], FasL [22], and TRAIL [23]. Stromal cells in the tumor microenvironment also utilize exosomes to transfer molecular contents that affect cancer initiation and progression. MiR-21 is delivered by exosomes derived from neighboring stromal cells in the omental tumor microenvironment, conferring the malignant phenotype and chemoresistance in metastatic ovarian cancer cells. In breast cancer, exosomes secreted by mesenchymal stem cells (MSCs) might reprogram tumor behavior by transferring their molecular contents. MiR-16 is enriched in MSC-derived exosomes and is partially responsible for the anti-angiogenic effect by targeting vascular endothelial growth factor (VEGF) [24]. As discussed above, exosomes regulate the tumor microenvironment by modifying the physiological state of the recipient cell [25]. Thus, exosomes are emerging as critical messengers in tumor progression and metastasis.

Long noncoding RNAs (lncRNAs) are operationally defined as transcripts of greater than 200 nucleotides that function without evident protein-coding function. LncRNAs are present in either the nucleus or cytoplasm, and they can interact with DNA, RNA or proteins [26]. Notably, lncRNAs were demonstrated to play important roles in multiple biological processes by directly or indirectly interfering with gene expression in various cancers [27]. For instance, H19 promotes tongue squamous cell carcinoma migration and invasion by targeting miR-let-7 [28]. The long noncoding RNA lnc-EGFR promotes hepatocellular carcinoma immune evasion by stimulating T-regulatory cell differentiation [29].

Interestingly, lncRNAs can also be packaged into exosomes and act as messengers in cell-to-cell communication [30]. Notably, some lncRNAs are enriched in exosomes, while others are barely present, indicating that some lncRNAs are selectively sorted into exosomes. Specific proteins might act as fundamental lncRNAs carriers to control lncRNA sorting into exosomes, though the exact regulatory mechanisms are not yet fully elucidated. Recently, exosome-derived long noncoding RNAs were reported to regulate tumor apoptosis [31], proliferation and migration and to induce angiogenesis [32]. These exosome-derived long noncoding RNAs have potential as diagnostic and prognostic biomarkers of various cancers [33]. The dysregulation of exosomal lncRNAs could affect the tumor microenvironment and regulate critical oncological behaviors [17]. For instance, hypoxic bladder cancer cells remodel the tumor microenvironment to facilitate tumor growth and development by secreting the oncogenic lncRNA-UCA1-enriched exosomes, and exosomal lncRNA-UCA1 in human serum has potential as a diagnostic biomarker for bladder cancer [34]. Thus, lncRNAs have gained increasing attention in exosome research.

In this review, we discuss the basic properties of exosomes and the functional roles of exosomes in cancer. In particular, we summarize the current knowledge regarding the contribution of exosome-associated lncRNAs to tumor microenvironments, such as tumor angiogenesis, tumor metastasis, and tumor drug resistance.

### Biogenesis and characteristics of exosomes in cancer

**Fig. 1:**
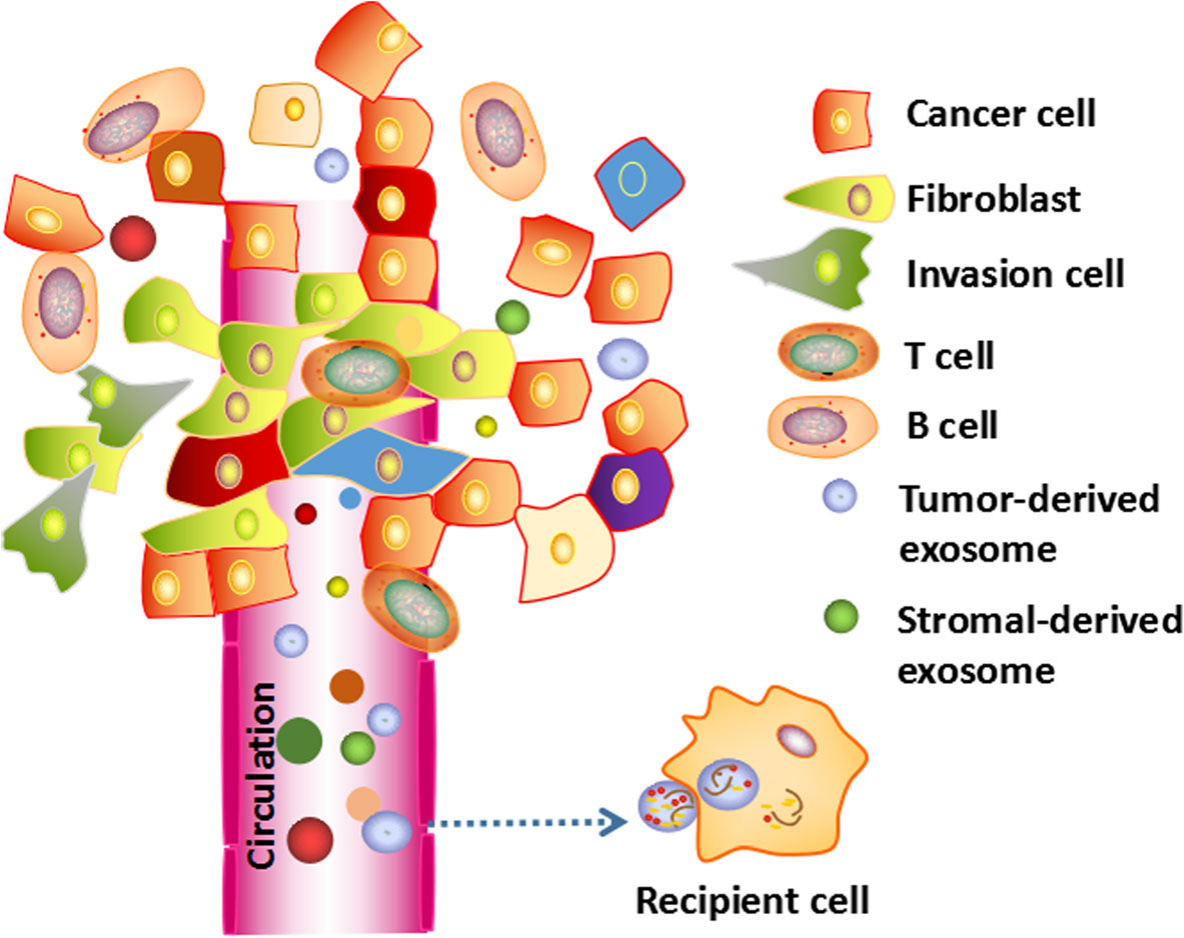
Exosomes were serected by cancer cells and stromal cells. Exosomes participate in cell-to-cell communications within tumor microenvironment. Circulating exosomes were tranfered to recipient cells, relesaing contents such as mRNAs, noncoding RNAs (miRNAs, lncRNAs and circRNAs), proteins, lipids, and metabolites

Extracellular vesicles (EVs) include apoptotic bodies, microvesicles, and exosomes. Exosomes emerged as important players in cell-to-cell communication in normal physiology and pathological conditions [35]. Exosomes are 40–150 nm EVs released by all cell types in the tumor microenvironment (Fig. 1) [36–38]. Exosomes, generated inside multivesicular endosomes or multivesicular bodies (MVBs), are secreted when these compartments fuse with the plasma membrane [39]. Exosomes likely remove excess and/or unnecessary constituents from the cells, functioning like garbage bags [40]. Additionally, exosomes modify the physiological state of the recipient cell via multiple mechanisms [41, 42]. On the one hand, exosomes directly target recipient cells through their surface molecules, inducing signaling via receptor-ligand interactions [43]. On the other hand, exosomes fuse with the membrane of a target cell, delivering their contents into its cytosol [44]. Exosomes display a particular organization and composition distinct from the parent cell, even though they share common features [45]. Emerging evidence suggests that exosomes in tumor microenvironments participate in facilitating tumorigenesis by regulating tumor angiogenesis, tumor immunity, and tumor metastasis [46].

### Exosome-associated long non-coding RNAs in cancer

Exosomes were confirmed to act as bridges for important information exchange between cells, carrying nucleic acids, proteins, and lipids to the recipient cells [47]. LncRNAs was verified to play important roles in cancer progression and metastasis. Interestingly, exosomes carry a broad range of lncRNAs, which are known to modulate gene expression by translational inhibition or by acting as competitive endogenous RNA [48–50]. We highlight the critical effects exerted by exosomal lncRNAs on tumor progression and drug resistance. For example, the exosome-associated lncRNAs Exo1–4 and RMRP are preferentially packaged and transmitted by the action of exosomes, resulting in enhanced recipient cell viability [46].

### Regulation of tumor microenvironment by lncRNAs in exosomes

**Fig. 2:**
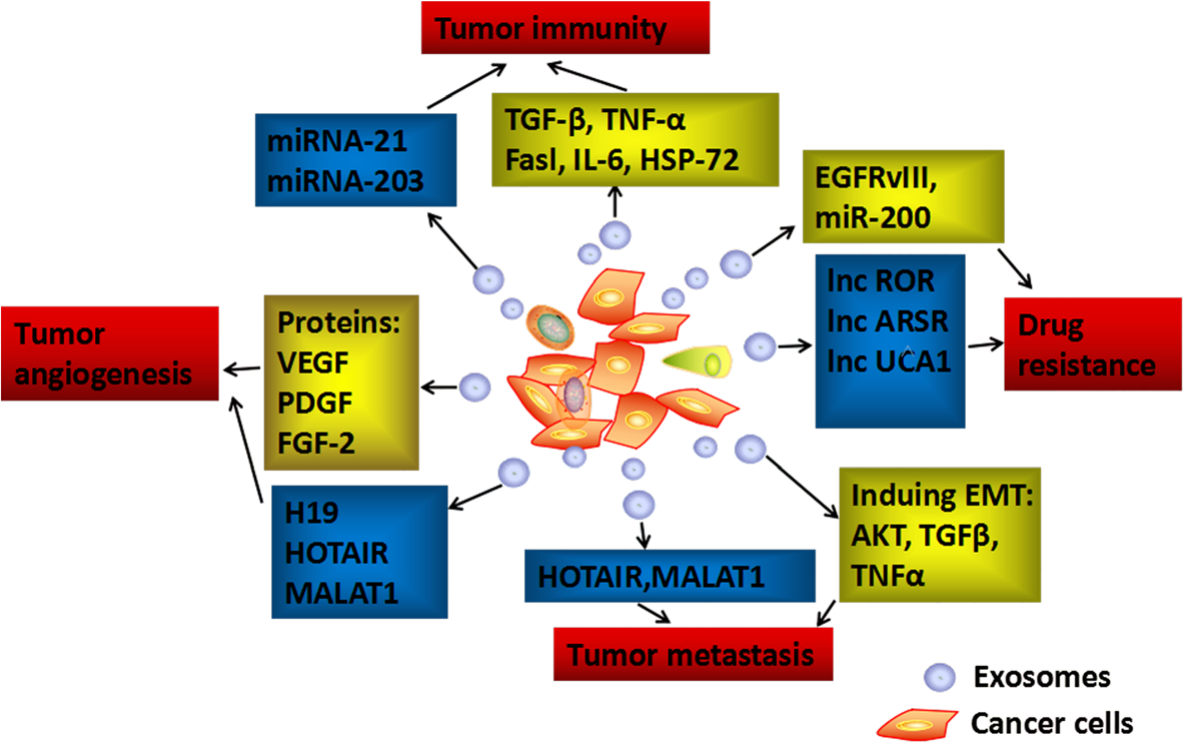
The functional role of exosomes in tumor microenvironment. Cancer cells and stroma utilize exosomes to modify surrounding cells within tumor microenvironment by transferring ncRNAs and proteins, which induce signaling via receptor-ligand interaction

Exchange of information in the tumor microenvironment can significantly affect tumor occurrence and development, as well as invasion, metastasis, and other malignant biological behaviors [35]. At the site of the primary tumor, tumor- and stroma-derived exosomes were verified to participate in regulating tumor proliferation and drug resistance [51]. Various mechanisms have been proposed [52]. Exosomes modulate the escape of cancer cells from immune cells by releasing immunoregulatory molecules, such as TGF-β [53], FasL [54], and HSP72 [55] (Fig. 2). lncARSR is highly expressed in sunitinib-resistant RCC cells, and cancer cells can disseminate survival skills to other recipient cells via exosomes containing lncARSR [47]. This revealed that exosomal lncRNAs, such as lincUCA1 [31] and lincROR [42], can exert critical functions in the cancer metastatic process (Fig. 2). In addition, tumor-derived exosomes can alter the cellular physiology of distant non-tumor cells to allow dissemination and growth of cancer cells. The influence of exosomal lncRNAs in the tumor microenvironment has recently been in the limelight [56, 57]. Exosome-mediated interactions between cancer cells and their surrounding cells within the tumor microenvironment contribute to tumor progression and metastasis (Fig. 2).

### Exosomal lncRNA and tumor angiogenesis

Tumor angiogenesis comprises several steps: enzymatic degradation of the vessel’s basement membrane, EC proliferation, migration, sprouting, branching, and tube formation. In the tumor microenvironment, exosomes released by different cell types have been shown to be important mediators during the process of tumor angiogenesis [58–60], such as mesenchymal stem cells, stromal cells, and endothelial cells [61–64]. LncRNAs secreted by tumor-derived exosomes could stimulate the proangiogenic potential of circulating angiogenic cells by increasing their expression of both membrane molecules and soluble factors [65].

For instance, the long non-coding RNA HOTAIR is highly expressed in glioma cells. Interestingly, HOTAIR is packed into exosomes secreted by glioma cells and conveyed to endothelial cells. Then, HOTAIR stimulates angiogenesis by increasing the expression of VEGFA [66], a well-known proangiogenic factor [67]. H19 has been closely associated with hepatocarcinogenesis [68], hepatic metastases [69] and angiogenesis [70]. In CD90^+^ liver cancer cells, lncRNA H19 is abundant and is packed inside exosomes. Furthermore, exosomal lncRNA H19 is transferred to and internalized by endothelial cells, promoting an angiogenic phenotype and cell-to-cell adhesion by upregulating VEGF production and release [71]. Similarly, lncRNAs HOTAIR and MALAT1 are upregulated in endothelial-derived exosomes by ethanol, which might have implications for alcohol-induced tumor angiogenesis [72–74].

### Exosomal lncRNA and tumor metastasis

Cancer-derived exosomes have been shown to participate tumor progression. Many groups have confirmed that tumor-derived exosomes are involved in the different steps of the metastatic cascade. Exosomes alter the cellular physiology of both surrounding and distant non-tumor cells to allow dissemination and growth of cancer cells, by triggering vascular permeability or by conditioning pre-metastatic sites in distant organs [75]. Interactions between metastatic cells and their microenvironment via lncRNA-containing exosomes have also been demonstrated. For instance, the lncRNA MALAT-1 is highly expressed in non-small-cell lung cancer (NSCLC) patients, and exosomal MALAT-1 is positively associated with TNM stage and lymphatic node metastasis. In lung cancer cell lines, serum exosome-derived lncRNA MALAT-1 promotes tumor migration and prevents tumor cells from apoptosing [76]. Meanwhile, HOTAIR, MALAT1 and MEG3 are significantly differentially expressed in exosomes isolated from cervical cancer patients compared to normal controls [77]. In bladder cancer, HOTAIR is packed into bladder cancer patient urinary exosomes and correlates with cancer progression. Conversely, loss of HOTAIR expression in urothelial bladder cancer cell lines inhibits epithelial-to-mesenchymal transition (EMT), which is responsible for tumor metastasis [78]. In colorectal cancer (CRC), exosomal lncRNA 91H is highly expressed in the serum of CRC patients and usually decreases after operation. Furthermore, lncRNA 91H was verified to promote tumor migration and invasion by regulating HNRNPK expression [79].

The same phenomenon has been demonstrated in gastric cancer (GC). ZFAS1 is elevated in GC cells, and high expression of ZFAS1 has been correlated with lymphatic metastasis and TNM stage. Interestingly, ZFAS1 is delivered by exosomes, enhancing GC cell proliferation and migration by promoting EMT [80].

### Exosomal lncRNA and tumor drug resistance

Drug therapy is the main means of cancer treatment. However, drug resistance is a thorny problem affecting the effectiveness of treatment. Exosomes, as an important communicator of intercellular communication, participate in drug resistance delivery. Tumor cells and stromal cells in the tumor microenvironment can secrete drug-resistant exosomes, containing proteins and non-coding RNAs. As a research hotspot in recent years, long non-coding RNAs secreted by exosomes play important roles in the transfer of tumor resistance.

**Fig. 3:**
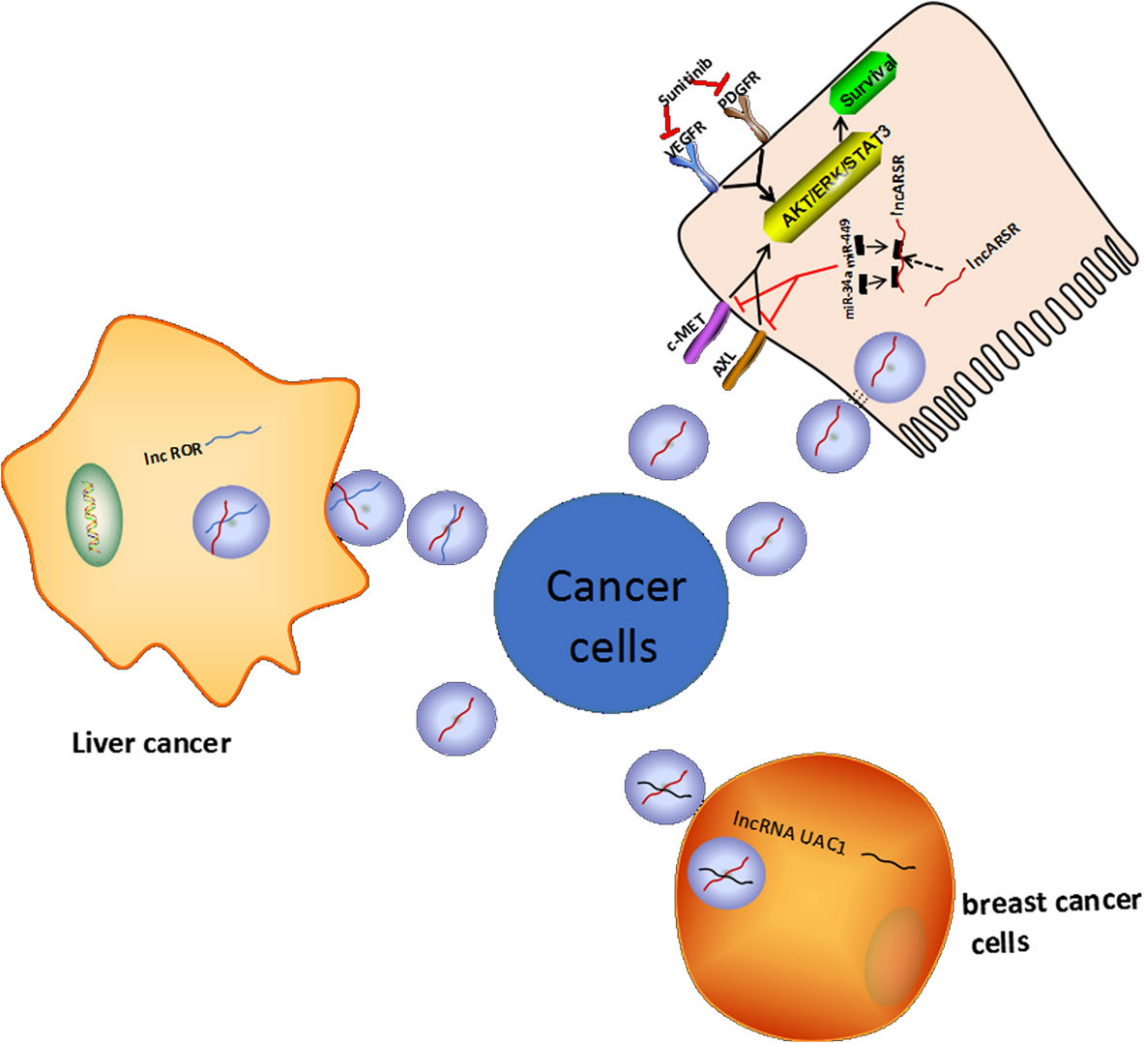
LncARSR can be packaged into exosomes and secreted from sunitinib-resistant RCC cells, transferring resistance to recipient-sensitive cells

Sunitinib resistance is a therapeutic problem for patients with advanced renal cell carcinoma (RCC). LncARSR is highly expressed in sunitinib-resistant RCC cells compared to sunitinib-sensitive RCC cells. LncARSR is packed into exosomes and taken in by recipient cells. Exosomal lncARSR competitively binds miR-34 and miR-449, leading to the increased expression of AXL/c-MET and reactivation of STAT3, AKT, and ERK signaling. Interestingly, activated AKT induces phosphorylation and degradation of FOXO1 or FOXO3a, resulting in the transcriptional derepression of lncARSR, forming a positive-feedback loop [81] (Fig. 3). Additionally, demonstrations of exosome-mediated intercellular pathways provide new insights into hepatocellular carcinoma (HCC) resistance to chemotherapeutic stress [82, 83]. Long non-coding RNAs are enriched within exosomes derived from HCC, such as CAR Intergenic 10, HAR1B, and DISC2 [42]. Linc-VLDLR is reportedly responsible for chemoresistance and is transferred to recipient cells in HCC, though the mechanism is still unclear [84]. TGFβ can reduce the sensitivity of HCC cells to sorafenib or doxorubicin, and the exosomal lncRNA ROR helps recipient cells acquire chemoresistance in HCC by activating the TGFβ signal pathway (Fig. 3). In estrogen receptor (ER)-positive breast cancer cells, lncRNA UCA1 is loaded in exosomes, resulting in tamoxifen resistance (Fig. 3) [31].

Above all, exosomal lncRNAs may cause acquired chemoresistance within tissues and contribute to a loss of therapeutic effect. The lncRNAs transmitted by exosomes can promote tumor cell adaptation to the tumor environment and the obtainment of survival skills [85, 86].

### Exosomal lncRNAs and characteristics of tumor stem cells

Cancer stem cells (CSCs) are a subpopulation of cancer cells that have the ability to self-renew and give rise to new tumors and metastases. To date, CSCs have been identified in many solid tumors, such as colorectal cancer, renal cancer, and hepatocellular cancers [87]. In addition to the aforementioned promotion of tumor immune escape mediated by exosomes, lncRNAs secreted by exosomes regulate tumor stem cell characteristics [88–90]. Intratumoral hypoxia is one of the most fundamental tumor microenvironment stresses for solid tumors. In the hypoxic microenvironment, tumor cells secrete exosomes containing lncRNAs. Through the regulation of exosomal lncRNAs, the proliferation ability of tumor stem cells is enhanced, thus promoting tumor proliferation and metastasis [34]. In hypoxic bladder cancer cells, hypoxia enhances exosome-mediated shuttling of lncRNAUCA1 into bladder cancer cells. Furthermore, lncRNA-UCA1 promotes tumor growth and progression by inducing EMT [91].

### Exosomal lncRNAs as new potential tumor biomarkers

**Fig. 4:**
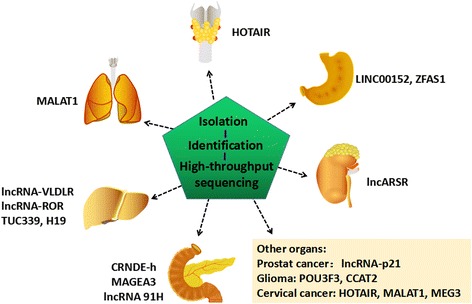
An increasing number of exosomal lncRNAs have been reported to be aberrantly expressed in human cancers. Exosomal lncRNAs may be potential biomarkers for cancers

With the demonstration of the effects of exosomal lncRNAs on tumors, circulating tumor-derived exosomes have emerged as promising biomarkers to monitor cancer progression, such as lncARSR and UAC1 (Fig. 4). Serum exosomal levels of several miRNAs are significantly higher in primary cancer patients compared with healthy controls, such as miR-21 and miR-125b [92, 93]. In addition to miRNAs, lncRNAs associated with tumor-derived exosomes are attractive as potential biomarkers. In colorectal cancer (CRC), MAGEA3 has been reported as a colorectal cancer-related serological biomarker [94]. Additionally, exosomal CRNDE-h levels has been reported to be highly elevated in CRC patients’ serum and significantly associated with factors of poor clinical outcome in CRC [95]. Moreover, high CRNDE-p and low miR-217 serum exosomal levels are correlated with advanced clinical stages (III/IV), tumor classification (T3/T4), and lymph node or distant metastasis [96]. Thus, combined evaluation of serum exosomal CRNDE-p and miR-217 levels shows diagnostic and prognostic potential for CRC patients. In gastric cancer (GC), LINC00152 is likely to be contained in exosomes. Plasma LINC00152 were significantly elevated in gastric cancer patients compared with healthy controls [97]. A similar study demonstrated that ZFAS1 was highly expressed in serum exosomes of GC patients, and upregulated ZFAS1 was significantly correlated with lymphatic metastasis and TNM stage. This revealed that exosomal ZFAS1 may serve as a potential diagnostic biomarker for GC [98]. Nonetheless, lncRNAs in exosomes can serve as biomarkers in multiple other cancers, such as lncRNA-p21 in prostate cancer and HOTAIR in bladder cancer [99]. As mentioned above, exosomal lncRNAs might have potential as biomarkers for cancer.

## Conclusions

Exosomes are a new means of exchanging information between cells, and play a significant role in tumor microenvironment. The rapid development of exosome research has elucidated novel mechanisms underlying the intrinsic intercellular communication networks during cancer initiation and progression [100, 101]. Tumor-derived exosomes promote angiogenesis and coagulation, modulate the immune system, and remodel surrounding parenchymal tissue, which together support tumor progression [102, 103]. Emerging evidence reveals that lncRNAs play significant roles in regulating the tumor microenvironment and tumor progression [104–106]. However, the physiological and pathological roles of exosomes and exosomal lncRNAs in the tumor microenvironment remain to be further explored. Meanwhile, the quantity and heterogeneity of exosomes in body fluids may be a drawback to their use as biomarkers, for these can lead to false negatives or positives in cancer diagnosis. To overcome these obstacles, we need to learn more about these delivery packets and their precise regulatory mechanisms in cancer progression. We believe that an in-depth understanding of exosomes in the tumor microenvironment may contribute to the design of cancer-diagnostic and cancer-prognostic tools. Effective therapeutic strategies for cancer using exosomes as drug carriers are expected in the near future.

Exosomes are detected in the tumor microenvironment, and exosomal lncRNAs play critical roles in facilitating tumorigenesis by regulating angiogenesis, immunity, and metastasis. In the future, circulating exosomal lncRNAs may be used as liquid biopsies and noninvasive biomarkers for the early detection, diagnosis, and treatment of cancer.

## Abbreviations

CRC: Colorectal cancer
CSCs: Cancer stem cells
EMT: Epithelial-tomesenchyme transition
EVs: Extracellular vesicles
GC: Gastric cancer
HCC: hepatocellular cancer
LncRNA: Long noncoding RNA
NcRNAs: Non-coding RNAs
NSCLC: Non-small-cell lung cancer
RCC: Renal cell carcinoma
TEX: Tumor-derived exosomes
